# Accounting of GHG emissions and removals from forest management: a long road from Kyoto to Paris

**DOI:** 10.1186/s13021-017-0089-6

**Published:** 2018-01-03

**Authors:** Joachim H. A. Krug

**Affiliations:** Silviculture and Forest Ecology of the Temperate Zones, Georg-August-Universität Göttingen, Büsgenweg 1, 37077 Göttingen, Germany

**Keywords:** Forest management accounting, Kyoto Protocol, Paris Agreement, Nationally Determined Contribution (NDC), Incentives

## Abstract

**Background:**

Forests have always played an important role in agreeing on accounting rules during the past two decades of international climate policy development. Starting from activity-based gross–net accounting of selected forestry activities to mandatory accounting against a baseline—rules have changed quite rapidly and with significant consequences for accounted credits and debits. Such changes have direct consequences on incentives for climate-investments in forestry. There have also been strong arguments not to include forests into the accounting system by considering large uncertainties, procedural challenges and a fear of unearned credits corrupting the overall accounting system, among others. This paper reflects the development of respective accounting approaches and reviews the progress made on core challenges and resulting incentives.

**Main text:**

The historic development of forest management accounting rules is analysed in the light of the Paris Agreement. Pros and cons of different approaches are discussed with specific focus on the challenge to maintain integrity of the accounting approach and on resulting incentives for additional human induced investments to increase growth for future substitution and increased C storage by forest management. The review is solely based on scientific publications and official IPCC and UNFCC documents. Some rather political statements of non-scientific stakeholders are considered to reflect criticism. Such sources are indicated accordingly. Remaining and emerging requirements for an accounting system for post 2030 are highlighted.

**Conclusions:**

The Paris Agreement is interpreted as a “game changer” for the role of forests in climate change mitigation. Many countries rely on forests in their NDCs to achieve their self-set targets. In fact, the agreement “to achieve a balance between anthropogenic emissions by sources and removals by sinks of greenhouse gases in the second half of this century” puts pressure on the entire land sector to contribute to overall GHG emission reductions. This also concerns forests as a resource for the bio-based economy and wood products, and for increasing carbon reservoirs. By discussing the existing elements of forest accounting rules and conditions for establishing an accounting system post 2030, it is concluded that core requirements like factoring out direct human-induced from indirect human-induced and natural impacts on managed lands, a facilitation of incentives for management changes and providing safeguards for the integrity of the accounting system are not sufficiently secured by currently discussed accounting rules. A responsibility to fulfil these basic requirements is transferred to Nationally Determined Contributions. Increased incentives for additional human induced investments are not stipulated by the accounting approach but rather by the political decision to make use of the substitution effect and potential net removals from LULUCF to contribute to self-set targets.

## Background

The United Nations Framework Convention on Climate Change (UNFCCC) from 1992 obliged its Parties to provide national inventory reports on greenhouse gas emissions. The question on whether and how to include biological sinks and sources in an accounting system was intensively discussed during the Third Conference of the Parties (COP3) to the UNFCCC in Kyoto in 1997—after the general commitments were set for other sectors. The provision to include biological sinks into national accounting (and by this potentially alleviate emission reducing commitments from other sectors) was received as concession to facilitate the Parties’ approval of the Kyoto Protocol (KP) and their national commitments [1]. Schlamadinger et al. described the inclusion of biological sinks as “… a negotiated solution produced by an evolving political process that had to deal with considerable scientific uncertainty” [2]. In detail, Art. 3.4 of the KP prompts to “…decide upon modalities, rules and guidelines as to how and which additional human-induced activities related to changes in greenhouse gas emissions by sources and removals by sinks in the agricultural soils and the land-use change and forestry categories shall be added to, or subtracted from, the assigned amounts for Parties included in Annex I,…” [3]. With Art. 3.4, the opportunity to consider emissions and removals from forest management in the national emission reduction commitment was established.

However, this agreement was elaborated “… under time pressure and without the support of a fully considered scientific basis” [2]. And more specifically, the KP did not provide specific rules as on how emissions and removals from Land Use, Land Use Change and Forestry (LULUCF) were to be incorporated into the general accounting system. Thus, the Subsidiary Body for Scientific and Technological Advice (SBSTA) of the UNFCCC was mandated to work out respective rules and procedures, and the Intergovernmental Panel on Climate Change (IPCC) was invited in 1998 to elaborate a Special Report on LULUCF to support the development of these rules [2]. Two years later, this Special Report defined options and terms for LULUCF accounting and the inclusion of additional activities under Art.3.4 of the KP [4]. It also discussed related challenges and conditions—however, no defined single procedure on accounting LULUCF emissions and removals was elaborated. The Marrakesh Accords, adopted by the COP11/MOP1 of the UNFCCC in Montreal in 2005, suggested the need for improved scientific understanding in support of the development of effective climate change mitigation policies as well as for adequate reporting of progress achieved in reducing GHGs emissions by the Parties to the Kyoto Protocol [5]: Marrakesh Accords decision 11/CP.7 on LULUCF invited the Intergovernmental Panel on Climate Change (IPCC) to “develop practicable methodologies to factor out direct human-induced changes in C stocks and greenhouse gas emissions by sources and removals by sinks from changes in C stocks and greenhouse gas emissions by sources and removals by sinks due to indirect human-induced and natural effects [such as those from carbon dioxide (CO_2_) fertilization and nitrogen (N) deposition], and effects due to past practices in forests (pre-reference year), to be submitted to the Conference of the Parties at its tenth session”. Definitive accounting options for LULUCF were provided by the so-called “Accra Accounting Options” assembled in Accra (Ghana, 21–27, August 2008) by the convened delegates of the Ad Hoc Working Group on Further Commitments for Annex I Parties under the Kyoto Protocol (AWG KP) and reflect the advisable accounting methodologies discussed at that time [6, 7].

Most important reasons to not define one specific accounting procedure for the LULUCF sector (contrary to other sectors) were the recognition of complexities inherent to LULUCF activities [2], high levels of reporting uncertainties, very different pre-1990 conditions among Parties (so-called “national circumstances”) and to avoid unearned “windfall credits” potentially corrupting of the entire accounting system. To avoid undermining the, at that stage, existing agreement, LULUCF accounting was limited, e.g. by a cap on accountable emission from forest management. As a consequence, also incentives for human-induced climate-investments in forestry were quite restricted, although the potential to increase of removals and to decrease of emissions for other sectors (e.g. by substituting energy-intensive products by wood-products) is an almost unique feature of the forest sector.

Criticism on the inclusion of biological sinks is also based on the argument, e.g. by the environmental stakeholder FERN that terrestrial emissions cannot be compared to fossil emissions: While fossil emissions are “irreversible for all practical purposes”, terrestrial carbon pools are “highly reversible” and hold a “natural limit” [8]—although this argument does not consider the substitution potential of terrestrial emissions. At that stage of the elaborations, it was also argued that terrestrial emissions are distributed over huge areas, with large inter-annual variations, they are “difficult to measure and nearly impossible to extrapolate” [8]. Scientific progress, however, reduced the importance of this argument. In its Special Report from 1998 the German Advisory Council on Global Change (WBGU) noted that the inclusion of terrestrial sinks “harbours some danger” because of the difficulties associated with verifying emissions from terrestrial carbon undermine the verifiability of overall reduction targets [1, 8]. WBGU also warned that the requirement of complicated accounting methodologies could lead to “the possibility of abuse” [1].

Since then, the accounting options have been subject to further elaboration, specifications and controversial discussions among experts. Substantial progress has been made in concern of uncertainties, estimating and reporting. Further progress must be recognized concerning the challenges identified by the Special Report on LULUCF [4]; however, a consistent and unchallenged approach has not yet been identified. Such an approach is expected to meet the following criteria [4]:

- Factor out indirect-human induced and natural effects;
- Consider national circumstances (e.g. age-class effects);
- Form a safeguard for environmental integrity;
- Provide incentives to adapt forest management practices; and
- Represent robust and transparent accounting procedures.

This review provides an overview on the development of accounting rules for LULUCF since the KP and the challenges ahead for the implementation of the Paris Agreement (PA). It focuses on the above mentioned criteria of the Special Report on LULUCF [4] with the goal to identify improvements and shortcomings to potentially contribute to the on-going negotiations. The review is limited to accounting and resulting consequences on the integrity of the accounting approach and to incentives for increasing climate-investments in forestry. Reporting challenges are beyond its scope.

## Main text

### The history of accounting rules

#### Voluntary gross–net accounting with cap (Kyoto Protocol, first commitment period)

For the first commitment period of the KP (2008–2012), the accounting options were summarised by the Accra Options [6]. The Accra options reflected discussions about mandatory or voluntary accounting of KP Art. 3.3 (afforestation, reforestation and deforestation: ARD) and Art. 3.4 (forest management, FM; grassland management, GM; cropland management, CM; and revegetation, RV), gross**–**net accounting and an upper limit by a cap or a discount factor, net–net accounting, a forward-looking baseline, and land-based net-net accounting of Art. 3.3 and Art. 3.4 under the convention reporting land categories (compare [7]). These accounting options applied for Annex I Parties to the Kyoto Protocol (per definition “Developed Nations and Nations with Economies in Transition”) with emission limitation and reduction commitments. Non-Annex I Parties could be involved by specific “mechanisms”, with again specific rules and procedures.

While accounting of emissions from ARD (KP Art. 3.3) was mandatory for Annex I Parties, accounting of FM, GM, CM and RV (KP Art. 3.4) was voluntary—whereas it became mandatory for a second commitment period when it was elected in the first. In any way, every Party must deliver annual reports on LULUCF emission (i.e. CO_2_, CH_4_ and N_2_O, by the pools above- and below ground biomass, deadwood, litter and soil carbon). That National Inventory Reports (NIR) were again subject to specific regulations and are to be continuously improved.

The voluntariness of KP Art. 3.4 was to consider risks and challenges linked to the LULUCF sector. On the one hand, the LULUCF sector provides opportunities to reduce greenhouse gases (CO_2_ as well as non-CO_2_ gases), to enhance terrestrial C stocks, to provide renewable energy, to substitute fossil-carbon-intensive products and to conserve existing C stocks [2]. On the other hand, however, the sector and, i.e. FM could also provide meaningful sources of greenhouse gases. Emissions from deforestation and forest degradation are obvious, but current forests can also be unstable and turn into sources, e.g. when affected by calamities, potential saturation effects (e.g. ageing forests with smaller annual sequestration rates than before), and a rather slow responding ability.

Further on, since different Parties were facing quite different conditions, especially age classes of forests and risks of fire, the option to account for FM or not, as well as the decision to apply gross–net or net–net accounting, was kept open. This allowed single Parties to select more favourable options to consider “national circumstances”. For example, Germany or Austria, both Parties with substantial forest resources and sustainable management for centuries, were facing age-class structures that provided meaningful sinks in 1990. These structures are originating, among other reasons, from substantial reforestation activities in the beginning and mid-twentieth century (i.e. following reparation harvests, compare [9]). Those forests matured till the turn of the century: due to harvesting activities and reduced annual growth rates in higher ages. These stands have the tendency since 1990 to continuously reduce sequestration rates. Under net-net accounting, this circumstance (defined as “age-class legacy” when emissions are resulting from pre-1990 activities) would be calculated as considerable emissions from FM.

Moreover, when accounting is based on a comparison with a single base year it may create large net-credits or net-debits for a Party simply because the base year net-emissions happened to be much smaller or much bigger than those observed during the commitment period, a choice which could be best described as a “base year lottery” among different parties [10]. Also, potentially high internal variability arising from natural disturbances could result in erratic impacts. This contradicts the idea that accounting is supposed to reflect the impacts of human-induced actions, only. Moreover, legacy effects can mean that two countries with similar management practices may have very different accounting consequences [10, 11]. These circumstances could justify a special role of the forestry sector when its rather slow responding ability is considered—otherwise, comparable circumstances may concern other sectors as well. Germany, for example, benefits from “windfall-credits” by the close down of inefficient and far out-dated industries in the former German Democratic Republic after the 1990-reunification.

Further on, the influence of natural effects beyond direct human control (e.g. droughts, changed precipitation patterns, prolonged vegetation periods, enrichment of atmospheric CO_2_ and N depositions), defined as natural and indirect human-induced impacts on growth, were to be excluded from accounting. In order to enable an accounting approach for forest management, it was needed to distinguish between “direct human-induced contributions” and “natural” or “indirect human-induced contributions” to the overall C stock change and GHG emissions of forest ecosystems [12]. The Marrakesh Accords decision 11/CP.7 on Land Use, Land-Use Change and Forestry invited the Intergovernmental Panel on Climate Change in 2001 to “develop practicable methodologies to factor out direct human-induced changes in C stocks and greenhouse gas emissions by sources and removals by sinks from changes in C stocks and greenhouse gas emissions by sources and removals by sinks due to indirect human-induced and natural effects [such as those from carbon dioxide (CO_2_) fertilization and nitrogen (N) deposition], and effects due to past practices in forests (pre-reference year), to be submitted to the Conference of the Parties at its 10th session” [13]. However, the IPCC meeting on “current scientific understanding of the processes affecting terrestrial carbon stocks and human influences upon them” resumed in 2003 that the “scientific community cannot currently provide a practicable methodology that would factor out direct human-induced effects from indirect human-induced and natural effects for any broad range of LULUCF activities and circumstances” [14]. Efforts have been undertaken to partition between the effects, e.g. through combining inventory data and biophysical tree growth models [15]. However, such factoring out remained an academic exercise and the managed land proxy was agreed on as compromise between parties: Emissions and removals on managed land are assumed occurring by direct human induced impacts only—and unmanaged lands are not reported [16]. The obvious shortcoming of this compromise is the circumstance that natural and indirect human-induced impacts on C stock changes of managed land are still among the reported emissions. The application of net-net accounting and capping or discounting of accountable emissions was proposed, both approaches are reducing the impact of those undesired emissions. However, while a cap also limits incentives to increase ambitions for increased removals, a discount factor supports a maintained incentive—but increases the costs (ambitions or efforts required) from the beginning.

As a result, the variety of accounting options did lead to a situation, where the decision for or against a specific accounting option could result in a far more vital impact on accountable emissions and removals than the actual emissions and removals from FM themselves. This came true, e.g. for Germany with a meaningful sink from FM which has been declining since 1990. This sink-reduction was calculated at about 17,361 Mt CO_2_e during the first commitment period [4, 9, 17, 18]: Under net–net accounting, this amount would have been debited to the overall national commitment. Under gross–net accounting the still existing sink of 58,179 Mt CO_2_e was accounted for, although this credit was capped at 4547 Mt CO_2_e: Debits or credits from forest management activities are subject to a country-specific cap, listed in the appendix to the Annex of Decision 16/CMP.1 [19]. Setting the size of the cap for each country was informed by 3% of the base year emissions or 15% of the forest management sector, which ever was less. It must be noted that the reference of a cap for FM to a Party’s overall base year emissions holds not logical ground and can be perceived as a political decision. Thus the Party’s overall emissions determine the volume of accountable emissions and removals by FM.

The objective pursued by the cap was to exclude non-human impacts (windfall profits as well as compliance risks) on LULUCF by limiting accountable credits and debits from gross–net accounting (and to limit credits potentially gained from the joint-implementation mechanism [20]). Another argument for constraining gross–net accounting could be to avoid a ‘too easy’ compensation of other sectors’ emissions by Parties which hold vast forest resources. However, the cap introduced limits to accountable credits that do reflect purely political decisions in order to provide for national circumstances (Decision 16/CMP.1, Appendix Z [19]) and can be interpreted as volition to attach more or less importance to the land-use and forestry sector. Concessions for national circumstances were explicitly provided for Parties whose ratification of the KP was considered as indispensable, like Russia or Japan [20].

The LULUCF accounting rules for the first commitment period received a number of justified criticisms, referring to the high complexity, and the result that they do not provide sufficient incentives in the forest sector since the forest management cap is typically smaller than the current sink [21]. Further on, it was politically criticised that environmental integrity (in terms of system integrity of the accounting approach) was not guaranteed “since most of the credits are obtained without additional efforts, and the voluntary choice of some activities may lead to unbalanced accounting (only activities yielding credits are included)” [21].

#### Mandatory accounting against a forward-looking baseline (Kyoto Protocol, second commitment period)

Preparations for accounting FM in a second commitment period of the KP (2013–2020) focussed on improved accounting rules and the consideration of additional activities (wetland drainage and rewetting) and pools (harvested wood products, HWP). Rules for the second commitment period of the Kyoto Protocol were finally agreed on in Doha (COP 18, Katar) on December 08, 2012. The main modifications in the LULUCF sector were:

- Accounting emissions (sinks and sources) from FM are mandatory;
- Emissions are accounted towards a reference level (most Parties developed a business-as-usual-projection: a forward looking baseline) as net-net accounting approach;
- Emissions resulting from natural disturbances (e.g. forest fire) can be excluded from accounting;
- Changes of C-stocks in harvested wood products (HWP) are accountable (and are not “instantly oxidised” as assumed in the first commitment period); and
- A new activity “wetland drainage and re-wetting” was introduced.

On this basis, the European Union decided on May 21, 2013 on accounting rules on greenhouse gas emissions and removals resulting from activities relating to LULUCF (Decision 529/2013/EU [22]). The decision provided accounting rules on a mandatory basis to the activities of afforestation, reforestation, deforestation (ARD) and forest management (FM), as well as for activities of grazing land (GL) management and cropland (CL) management. Further accounting rules were applicable on voluntary basis to revegetation (RV) and wetland drainage and rewetting activities (WL). More advanced, the accounting rules “should ensure that accounts accurately reflect human-induced changes in emissions and removals”, therefore emissions from ARD and “results from direct human-induced conversion of land (…) should be accounted for in their entirety” [22]. Thus, such emissions are to be accounted for in a gross–net approach. Emission from removals relating to GL, CL, RV and WL are to be accounted for by applying a base year to calculate changes in emissions and removals—which means a net-net approach.

Finally, emissions from FM, considering national circumstances, age-class structures and past as well as present management practises, should be accounted by the use of a reference level “to exclude the effects of natural and country-specific characteristics” [22]. Such reference levels should be “set transparently in accordance with Decisions 2/CMP.1 and 2/CMP.7”. The following elements were to be considered [23]:

- Removals or emissions by forest management as shown in greenhouse gas inventories and relevant historical data;
- Age class structures by using the latest available country specific inventory data;
- Forest management activities already undertaken;
- Projected forest management activities (under a business-as-usual scenario, considering silvicultural guidelines and excluding recent domestic policies);
- Continuity with the treatment of forest management in the first commitment period; and
- The need to exclude removals and emissions from accounting in accordance with Decision 16/CMP.1, paragraph 1 (factoring-out indirect human-induced and natural impacts on forest growth).

While it was and still is unsolved how to accurately factor-out removals from accounting in accordance with Decisions 16/CMP.1, it was assumed that the net-net accounting and an application of the cap could reduce meaningful impacts in this context [23].

On the other hand, the obligation to account emissions from FM increases incentives for climate-investments in forestry, but the cap (again politically determined without relation to a Party’s forest management ambitions) is again limiting such incentives.

Strong criticism of environmental stakeholders on the establishment and application of projected reference levels for FM were based on three aspects:

First, it was not transparent which period of within the “use of historic data” is to be applied to establish a reference for BAU. Thus, the application of a distinct period and its length could possibly result in a higher or lower reference level under the same BAU assumption. By this, the reference level could be prepared in a way which allows easier achievement and thus credits in future.

Secondly, the BAU projection could include emissions from harvesting for bioenergy. Thereby, these are not accounted for in the future as well [24].

Thirdly, any consideration of future harvesting levels potentially results in a reference level where credits can be gained when harvesting will be less than predicted. While a consideration of historical harvesting levels could rather undermine such un-earned credits, it must be recognised that most projected reference levels submitted for KP II incorporated predicted harvesting levels higher than historical ones [21, 23]. This would add on a principle uncertainty of a projection.

The pool of HWPs was added under “Production Approach” to the previously reportable pools above-ground biomass, below-ground biomass, litter, dead wood and soil organic carbon. Under the Production Approach, Parties are to include in their reporting exported products that are manufactured within the country of wood harvest. Specific regulations on HWPs described reportable categories (paper, wood panels and sawn wood, all to be considered with first order decay functions of default half-life values) and exclusions (like HWP from deforestation, products harvested for energy purposes etc. are reported on the basis of instantaneous oxidation). Especially the consideration of HWPs and their mitigation potential by material or energetic substitution is contradictorily debated: For example, the drafted “Klimaschutzplan 2050” of the German Federal Government (“climate protection plan”, a basis for National Determined Contributions (NDCs), prepared for COP 22 by the Federal Ministry for the Environment, Nature Conservation, Building and Nuclear Safety), did not even mention a mitigation potential by HWPs [25], while the Federal Ministry of Food and Agriculture and its Scientific Advisory Boards on Forest Policy and on Agricultural Policy, Food and Consumer Health Protection explain in their report on climate change mitigation in agriculture and forestry the explicit impact and magnitude of the related mitigation potential [26]. It can also be observed that lobby groups of the environmental sector justify a respective consideration or neglect of HWPs rather on ideological than scientific backgrounds [27]. The final version of the German Klimaschutzplan 2050, however, considered HWPs according to the EC’s recommendations [28, 29]. The inclusion of reportable HWPs under Production Approach can be expected to support improved material and energetic substitution effects. While accounting HWPs under FM is limited by the cap, substitution effects are not. A cascading utilisation of long-lasting wood products for material and energetic substitution, for example, can contribute to reduction targets of other sectors, and by this, might have an impact on adapted forest management schemes and incentivise climate-investments.

### Accounting post-2030

#### What the Paris Agreement requires

The Paris Agreement (PA, adopted on 12.12.2015 at COP 21) provided rather guidelines than specifications. All nations (no distinction between Annex I and non-Annex I, developed or developing countries, no KP-mechanisms any more) were legally bound to prepare Nationally Determined Contributions (NDCs); no levels of such contributions were defined. It was the goal that these individual contributions will be sufficient to achieve carbon neutrality in 2050: This implies that by 2050 all emissions are balanced by removals, as by forests. Since it is impossible to balance all current emissions by net removals from forestry, those emissions can only be reduced as far as possible and partly compensated by substitution effects from LULUCF and in a smaller extent by net removals from the forest sector.

However, no defined rules on what aspects of LULUCF (i.e. categories, activities, pools, etc.) need to be accounted, how they are accounted and which methods are to be applied [30], were established. It was stated that “Parties shall account for their Nationally Determined Contributions” and to provide “information necessary to track progress made in implementing and achieving its Nationally Determined Contributions” [31]. Thus it is understood that the member states are to design their own accounting system individually as long as it is compatible with its NDC and consistent to IPCC guidelines [30]. An ad-hoc working group (AWG-PA) is to develop further guidance for the NDCs by 2018.

In any case, it is understood that reporting and accounting will continue under the PA and follow the previous IPCC rules. It is important to recognise that the PA is independent to KP and the LULUCF accounting rules under KP, thus “the opportunity exists for a thorough analysis of the existing systems in place under KP, as a basis for developing an improved and simpler accounting system for LULUCF for post 2020” [30].

#### An interims solution: LULUCF accounting rules proposed by the European Commission

The European Parliament proposed a regulation on the inclusion of greenhouse gas emissions and removals from land use, land use change and forestry into the 2030 climate and energy framework [28] in July 2016. It “mirrors the coverage of the existing EU legislation for Member States under the Kyoto Protocol (529/2013/EU)”. The mandatory scope is in essence forest land and agricultural land, and land for which the use has changed from or to these uses. The approach proposed discards the parallel Kyoto Protocol reporting framework and streamlines the system with the UNFCCC “land-based” reporting framework. The scope includes greenhouse gases CO_2_, CH_4_ and N_2_O” [28]. The regulation further minimises the commitment of each Member State’s LULUCF sector to just have no net emissions, which is criticised as being unambitious by NGOs [32].

Further proposals follow the 2013 decision on LULUCF accounting (Decision 529/2013/EU [22]) to a far extent:

- ARD remains mandatory under GNA, emissions from activities on GL, CL, revegetation and WL are to be accounted voluntarily under NNA in reference to the base period 2005–2007. Reporting periods are 2021–2025 and 2026–2030;
- The same reporting periods are to be applied for Forest Management; the reference level is to be established transparently and in consideration of the business-as-usual procedures of the period 1990–2009;
- Limitations of accountable removals from FM are capped by 3.5% of the Member State’s base year emissions as under KP II; and
- Also the accounting approach for HWP and for natural disturbances remain “essentially unchanged compared to the Decision 529/2013/EU” [28].

It must be recognised that especially the determination of the period 1990–2009 for BAU-establishment could be rated to be an improvement: It limits opportunities to select “suitable” periods for BAU calculations. This could otherwise result in a pre-determined reference level which could potentially allow an easier accounting of credits. However, since a reference level allows an individually specified amount of emissions to be unreported, a situation may arise where emissions from forest management are mostly not reported—while removals are [33]. This could risk the integrity of the accounting system, e.g. when LULUCF removals are to compensate emissions in other sectors.

On October 13, 2017, the European Parliament’s plenary adopted the “LULUCF Regulation” which, among minor amendments, increased the cap to 7% (including a specified consideration of net removals from wood panels, sawn wood and dead wood) and changed the overall reference period from 1990–2009 to 2000–2012 [34]. Especially the changed (and shortened) reference period might receive criticism since it could include emissions from more recent policy changes (like increased used of wood for bioenergy) and could exclude emissions from the period 1990–2000 in which bioenergy-policies played a minor role. In this view, the projected reference level could incorporate emissions from bioenergy-harvests, while an emission reduction in the energy sector by the use of wood products for bioenergy would be accounted for. Such a situation would contradict the integrity claim of accounting, again. The enlarged cap, however, does incentivise for more climate-investments in FM since these can be accounted to a larger extent.

Still, it must be considered that the PA did not specify any accounting rules, and more specified guiding could be expected by the AWG-PA in 2018 only. However, the proposal of the European Commission and the European Parliament’s LULUCF Regulation assume the application of previously agreed-on rules. Major critics of political stakeholders focus on the low level of ambitions under FM (“no debit rule”), which is supported by the establishment of a reference level. The “no debit rule” simply states that the EU as a whole and for each member state, accounted emissions should not exceed accounted removals [35]. CAN also proclaims that unaccounted emissions from FM and a potential risk that individual RL would also be “inherently unreliable and can easily be exaggerated” [36], provide basis for debates [37]. As a scientific stakeholder of the debate, the European Forest Institute warns that “The definition of the reference scenario has a strong influence on the outcome of assessments” and recommends a well-founded justification of those [38]. The “mixed approach” (GNA for ARD; NNA for CL, GL and WL; and a BAU-RL for FM) is also criticised to be problematic due to the lack of comparability [31, 33].

#### The role of forests in NDCs

So far, the LULUCF sector, and especially forest management accounting, played an important but secondary role in the overall accounting and reporting system. Some arguments even suggest excluding biological sink from the overall accounting approach [1, 8]. The PA’s focus on NDCs, and the goal to achieve carbon neutrality in 2050, changes that perception fundamentally. It pushes forests especially, as potential sink and envisaged emission reductions from reduced deforestation, into a key role in meeting climate targets in many countries [39]. While the LULUCF sector contributed to an estimated 21–24% share to the anthropogenic greenhouse gas emission in 2010 [40, 41], a comprising analysis of the NDCs submitted in 2015 reveals that “LULUCF could provide about a quarter of total emission reductions planned” in 2030 [39]. A main impact is recognised by a steep reduction of emissions from deforestation in Brazil and Indonesia (combined with a reduction of peat fire emissions) and Russia. Russia is especially looking forward to an increasing sink in managed temperate and boreal forests. According to Grassi et al., these three countries are the most relevant countries in terms of the magnitude of the LULUCF contribution [39]. In a comparable conclusion, Forsell et al. also highlight the importance of the LULUCF sector to meet NDC targets [42]. The authors expect the LULUCF sector to contribute at a global level as much as 20% of the full mitigation potential of all the conditional and unconditional NDC targets.

In a similar understanding, the obligation to prepare NDCs and the implication that all emissions are to be balanced by removals in 2050 further emphasises the importance of REDD+ (Reducing Emissions from Deforestation, forest Degradation, and other forest activities). Although the relationship between financially supported REDD+ activities and NDCs remains uncertain (especially when different accounting and baselines are applied), it is remarkable that the PA “calls explicitly for all countries to make use of a full range of land-based mitigation options, and to take action on REDD+” [39]. This underlines the interpretation of the Paris Agreement as a “game changer” for the role of forests in climate change mitigation [39] and incentivises climate-investments in forest management, more then before.

However, recognition of a greater focus on the LULUCF sector must not ignore the challenges and unsolved issues regarding accounting. Still, there is a large scientific challenge of providing a practical methodology to factor-out direct human-induced mitigation action from indirect and natural effects [39, 42, 43] and to ensure the accounting system’s integrity [21]. The situation that also the PA provided rather guidelines than specifications on accounting results in different approaches on how to manifest mitigation targets in national NDCs. Grassi et al. identify four “cases” when classifying 68 countries’ NDCs in the table below:

**Table:** Overview on 68 submitted NDC accounting approaches by a classification of four “NDC cases”, modified from Grassi et al. [39]

“Case”	Type of mitigation target	Inclusion of LULUCF within the NDC	Countries with enough LULUCF information for this classification
1	Absolute target relative to base year	LULUCF to be treated as any other sector	Brazil, USA
2	“Conditional” (linked to financial, technical, or capacity-building support) and “unconditional” reduction targets relative to BAU scenario		Angola, Benin, Cambodia, Central African Republic, Chad, Colombia, Congo, Democratic Republic of Congo, Ecuador, Ethiopia, Gabon, Ghana, Guyana, Indonesia, Laos People’s Democratic Republic, Kenya, Madagascar, Malawi, Mali, Mexico, Morocco, Namibia, Senegal, Uganda, Zambia
3	Absolute target relative to base year	Special accounting rules	Australia, Canada, EU, Japan, Kazakhstan, New Zealand, Norway, Russian Federation, Switzerland, Turkey, Ukraine
4	Intensity target (relative to GDP)	Various approaches	Chile, China, India

The above table reveals an additional dimension to the question on how different accounting rules support incentives to invest in forest management changes: Next to a potential amount of accountable credits or debits from FM and to the impact of material and energetic substitution to other sectors, the inclusion of LULUCF within NDCs also depends on the type of the NDC’s mitigation targets. It is obvious that ambitions in LULUCF receive different incentives when mitigation targets are related to a base year, are conditional or are valued in relation to an overruling intensity target (e.g. case 4 in the above table).

#### Core challenges for forest management accounting

A constructive progress must be recognised in the development and approaches for forest management accounting. Especially the goal to achieve carbon neutrality in 2050, as agreed on in the PA, can be expected to further encourage the implementation of adapted forest management and a reduction of forest degradation and deforestation. However, most of the methodological challenges considered in the early discussions on forest management accounting remain unsolved.

Accounting rules necessary to incentivise human action for achieving a balance between anthropogenic emissions by sources and removals by sinks of greenhouse gases in the second half of this century must provide solutions to the following core challenges:

1. *Transparency and comparability between different countries* It should be feasible to convene results and consequences of different accounting options (potentially reflecting national circumstances) to an overall accounting system. The currently proposed differing NDC-approaches do not really deliver comparable contributions since the inclusion of LULUCF is based on differing approaches.
2. *Adequate reflection of management impacts* Especially the definition of a reference scenario has a strong influence on the outcome of assessments [38] which could even overrule actual management impacts [7]. For example, an inclusion of anticipated increased harvesting levels for the production of bioenergy (as potentially achievable by a suitable reference scenario) would cause a gross violation of the meaning of reference levels in reflecting BAU activities. Adequate guidelines are required to ensure that a decision on methodological aspects cannot master the actual impact of accountable emissions and removals from FM. Furthermore, definitions of reference levels require a well-founded and transparent justification to avoid a “base year lottery” [10].
3. *Safeguarding of the accounting system’s integrity* Since a reference level allows an individually specified amount of emissions to be unreported, the consequence may arise where emissions from forest management are not reported below a defined level—while removals are [33]. Moreover, integrity is not guaranteed when credits are obtained without additional efforts, i.e. when a “comfortable” baseline is selected for the establishment of a reference level, or when the reference level considers increased harvesting levels (e.g. in favour of bioenergy) [35]. Further on, the voluntary choice of some activities (like emissions from activities on GL, CL, revegetation and WL) may lead to unbalanced reporting when only activities yielding credits are included [21]. Thus appropriate accounting rules require respective safeguards to ensure the accounting system’s integrity on a stronger level.
4. *Factoring-out the direct human-induced impact on FM* A fundamental challenge which was identified in the early days of forest management accounting still remains valid. Various proposals also exist to factor-out direct human-induced impacts from indirect human-induced and natural impacts [5, 11, 15], scientific approaches could not solve it [14, 43] and it remains a methodical challenge [39]. A last IPCC revision on the use of the managed land proxy in 2010 too did review a number of proposed alternatives, but decided that these require further development [16]. A shortening of the time period between the baseline used for reference level establishment and the actual commitment period is at least limiting the potential magnitude of unjustified emissions and removals from indirect human-induced and natural impacts. In fact, this approach would establish comparable levels of undesired impacts in the reference period as well as in the commitment period, and by this, “neutralise” them [43]. The application of a dynamic baseline (representing BAU and accounting only “isolated” changes in management) could even further reduce those unjustified impacts [11].
5. *Incentives to adapt forest management practices* It is a core purpose of a RL that countries that do not change land management relative to BAU get neither debits nor credits. This is especially important to prevent countries from claiming already existing BAU sinks. Only activities that lead to reduced sources or enhanced sinks relative to BAU can claim credits if accounting in based on BAU. To support such activities, it must be considered that any capping or discounting of accountable emissions and removals automatically reduces incentives or even increases the price for additional efforts. On the other hand, it is conceivable that countries with vast reserves on managed forest land (or a deceiving reference level) could contribute too many or too easily accountable removals to the overall accounting system and thus potentially corrupt it by accounting unearned credits. Thus, it remains a challenge to provide incentives as supported by the goal of achieving a balance between emissions and removals by 2050, while ensuring integrity of the accounting system.

## Conclusions

It can be summarised that the LULUCF sector, and especially forest management, are expected to gain an increased attention, following the decisions of the PA. Forest management might even inherit a key role in meeting climate targets of NDCs [39], although it must be considered that LULUCF can at best contribute to emission reductions, but not facilitate climate neutrality. However, the challenges of LULUCF accounting specified for the first commitment period are still unsolved: First of all, it was not succeeded to establish an accounting approach that is based on a “practicable methodology that would factor out direct human-induced effects from indirect human-induced and natural effects for any broad range of LULUCF activities and circumstances” [14]. Secondly, the voluntarily application of different accounting options for different pools could allow individual member states to choose individually-favourable approaches. This could result in un-earned windfall profits. Thirdly, since the establishment of a reference level could allow pre-determined results to some extent and could include increasing harvesting levels (e.g. for bioenergy demands), an integrity of the accounting approach is not ensured.

Recalling the major reasons for not defining one specific accounting procedure for the LULUCF sector in the first commitment period under KP (high complexities of the activities, high levels of uncertainties, national circumstances, an avoidance of windfall profits, and the challenge to factor out direct human-induced effects from indirect human-induced and natural effects), it can be concluded that the accounting procedures developed allow some improvements. It should be considered that the lack of definitions and specific rules for reporting and accounting at this stage of negotiations served to reach an agreement among the Parties. However, since the PA and, e.g. accounting procedure proposed by EU in 2013, cannot define one specific procedure to solve the mentioned challenges, the responsibility to account for emissions and removals and to ensure integrity within the entire accounting system lies with the Parties, more than before.

And finally, it is expected that the LULUCF sector, and especially forest management, might gain high attraction in consequence of the PA. Thus it can be assumed that the enhanced focus on biological sinks in the preparation of NDCs may also encourage higher ambitions in research and support for forest management. However, it must also be recognised that the LULUCF sector can help but not solve the problem: When all NDCs submitted in summer 2016 are implemented in their full extent, a medial warming of 2.6–3.1 degrees Celsius by 2100 is still to be expected [44].

The question whether or not the challenges on LULUCF accounting are solved allows no straightforward answer. In consideration of the complexity of the LULUCF sector and manifold national circumstances, it should be acknowledged that the current rules and recommendations provide good ground for a compromise. On the other hand, it must also be considered that the core aim of accounting rules is not to provide incentives for improvements, but a framework for comparable standards. Increased incentives for additional human induced investments are not stipulated by the accounting approach, but rather by the political decision to make use of the substitution effect and potential net removals from LULUCF to contribute to self-set targets.

It is up to the member states to create incentives within their NDCs. This lays additional responsibility on the international community that needs to monitor and ensure the integrity of the NDCs.
